# Reporter Virus Neutralization Test Evaluation for Dengue and Zika Virus Diagnosis in Flavivirus Endemic Area

**DOI:** 10.3390/pathogens10070840

**Published:** 2021-07-03

**Authors:** Jannyce G. C. Nunes, Bruno T. D. Nunes, Chao Shan, Adriana F. Moraes, Tais R. Silva, Maria H. R. de Mendonça, Liliane L. das Chagas, Franco A. e Silva, Raimunda S. S. Azevedo, Eliana V. P. da Silva, Livia C. Martins, Jannifer O. Chiang, Livia M. N. Casseb, Daniele F. Henriques, Pedro F. C. Vasconcelos, Rommel M. R. Burbano, Pei-Yong Shi, Daniele B. A. Medeiros

**Affiliations:** 1 Department of Biochemistry & Molecular Biology, University of Texas Medical Branch, Galveston, TX 77550, USA; jannyce.gdcnunes@aluno.uepa.br (J.G.C.N.); brunonunes@iec.gov.br (B.T.D.N.); chshan@utmb.edu (C.S.); peshi@utmb.edu (P.-Y.S.); 2 Post Graduation Program in Parasitary Biology in the Amazon, Belém 66050-540, PA, Brazil; 3 Department of Arbovirology & Hemorrhagic Fever, Evandro Chagas Institute, Ananindeua 67015-120, PA, Brazil; adrianaf.moraes@hotmail.com (A.F.M.); taissilva@uol.com.br (T.R.S.); mariahelena.mendonca@uepa.br (M.H.R.d.M.); lilianechagas@iec.gov.br (L.L.d.C.); frankosilva@iec.gov.br (F.A.e.S.); raimundaazevedo@iec.gov.br (R.S.S.A.); elianapinto@iec.gov.br (E.V.P.d.S.); liviamartins@iec.gov.br (L.C.M.); janniferchiang@iec.pa.gov.br (J.O.C.); liviacasseb@iec.gov.br (L.M.N.C.); danielehenriques@iec.gov.br (D.F.H.); pedro.vasconcelos@uepa.br (P.F.C.V.); 4 Science and Health Institute, Pará State University, Belém 66113-010, PA, Brazil; 5 Biological Sciences Institute, ICS, Federal University of Pará, Belém 66050-000, PA, Brazil; rommel@ufpa.br

**Keywords:** reporter virus, neutralization test, Dengue, ZIKV, flavivirus, diagnosis

## Abstract

Reporter virus neutralization test (RVNT) has been used as an alternative to the more laborious and time-demanding conventional PRNT assay for both DENV and ZIKV. However, few studies have investigated how these techniques would perform in epidemic areas with the circulation of multiple flavivirus. Here, we evaluate the performance of ZIKV and DENV Rluc RVNT and ZIKV mCh RVNT assays in comparison to the conventional PRNT assay against patient sera collected before and during ZIKV outbreak in Brazil. These samples were categorized into groups based on (1) acute and convalescent samples according to the time of disease, and (2) laboratorial diagnostic results (DENV and ZIKV RT-PCR and IgM-capture ELISA). Our results showed that DENV Rluc assay presented 100% and 78.3% sensitivity and specificity, respectively, with 93.3% accuracy, a similar performance to the traditional PRNT. ZIKV RVNT_90_, on the other hand, showed much better ZIKV antibody detection performance (around nine-fold higher) when compared to PRNT, with 88% clinical sensitivity. Specificity values were on average 76.8%. Even with these results, however, ZIKV RVNT_90_ alone was not able to reach a final diagnostic conclusion for secondary infection in human samples due to flavivirus cross reaction. As such, in regions where the flavivirus differential diagnosis represents a challenge, we suggest the establishment of a RVNT panel including other flaviviruses circulating in the region, associated with the other serological techniques such as IgM ELISA and the investigation of seroconversion, in order to help define an accurate diagnostic conclusion using serology.

## 1. Introduction

Zika virus (ZIKV) is a mosquito-borne flavivirus (family Flaviviridae), isolated from the blood of a febrile sentinel rhesus monkey in the Zika Forest of Uganda in 1947 [1]. In the following 60 years, ZIKV was isolated from several species of *Aedes* spp. mosquitos [2,3] and associated to oligoassymptomatic cases, characterized by fever, rashes, myalgia, arthralgia, conjunctivitis, gastrointestinal disturbance, and headaches [4,5]. Since 2007, outbreaks have been reported in the Southeast regions of Africa and Asia [6,7,8]. Three ZIKV lineages are recognized and related in the epidemiologic data: East African, West African, and Asian lineages [9,10,11], but only one serotype has been found [8,10].

In 2015, ZIKV Asian lineage emerged in Brazil. A dramatic increase of microcephaly and Guillain-Barré Syndrome (GBS) cases have reported and associated with ZIKV [12,13,14]. Besides mosquito vectors, ZIKV was found to be transmitted by maternofetal route, sexual intercourse, blood transfusion, and organ transplantation [12,15]. Consequently, ZIKV spread quickly throughout South America and Caribbean region and a pandemic was established. In 2016, WHO announced “Public Health Emergency of International Concern” for ZIKV outbreak [16].

In these epidemic areas, with the co-circulation of several other flaviviruses, the diagnosis to differentiate them became a huge challenge. Since viremia is rather short-lived, to confirm the ZIKV infection the antibodies detection has been used [17] by ZIKV MAC-ELISA (CDC, Atlanta) or the IgM capture ELISA for ZIKV detection (InBios, Washington, USA) [16,18]. However, cross-reactive flaviviruses antibodies can make the interpretation of serological results notoriously difficult, especially in patients with previous flavivirus infections and/or vaccinations.

Plaque reduction neutralization test (PRNT) remains the gold standard to confirm and specify flavivirus antibodies, but it is labor-intensive, expensive, and not widely available, thus limiting its use for routine diagnostics, mainly during outbreaks [18,19]. One alternative test developed was the reporter virus neutralization test (RVNT). The ZIKV mCherry report virus has been used to evaluate ZIKV vaccine efficacy in mice and monkey models [20,21,22]. Recently, ZIKV and DENV-2 Renilla luciferase (Rluc) reporter assay was validated for human samples, using sera from asymptomatic travelers who returned from ZIKV epidemic areas. This reporter assay showed equivalent results as a traditional plaque assay and improved test turnaround time, dynamic range, and diagnostic throughput [23]. In this communication, we evaluated RVNT, using samples from ZIKV and Dengue symptomatic cases from Brazil, and also validated the ZIKV mCherry report virus for human samples.

## 2. Results

### 2.1. Groups’ Categories

We selected specimens from symptomatic patients compatible with DENV and/or ZIKV infections and tested by traditional PRNT and RVNT. The median age was 35.9 years old and 60% were female non-pregnant. These samples were categorized in four groups based on: (1) time of disease after symptoms’ onset and (2) laboratorial diagnosis results. Group I (*n* = 20) include patients who had diagnosis for DENV by RT-PCR and with samples collected between 1 to 5 days after symptoms’ onset; Group II (*n* = 19) include samples positives to DENV by IgM-capture ELISA and with samples collected with more than 5 days of symptoms’ onset. The patients with diagnosis for ZIKV were included in Group III (*n* = 19) and Group IV (*n* = 17). Group III contains acute samples tested for ZIKV RT-qPCR, while Group IV includes convalescent samples positives to ZIKV by IgM-capture ELISA from patients with diagnosis confirmed in the acute phase by ZIKV RT-qPCR. The majority of DENV positive samples in Groups I and II were collected before ZIKV introduction in Brazil [24], but five of them were collected during ZIKV outbreak, showing monotypic reaction to DENV in the serological tests (H815485, H817979, H817981, H819129, and H819519). On the other hand, ZIKV positive samples (Groups III and IV) were collected only during 2015/2016 outbreak (Table 1). 

### 2.2. Neutralization Tests

Eight samples from Group I probably have previous antibodies against DENV, but all of them were negative for ZIKV by PRNT_90_ and RVNT_90_. Therefore, in DENV secondary infection cases followed by other non-ZIKV flaviviruses (before ZIKV pandemic), observed in Group II, just a few samples showed unspecific antibodies against ZIKV and only in low dilutions for both assays. In Group III, despite the fact ZIKV genome was detected in theses samples, the ZIKV PRNT_90_ result was negative. In addition, eight samples (42.1%) were positive by both ZIKV and DENV RVNT_90_. These data indicate that in the scenario of a previous flavivirus infection followed by a ZIKV secondary infection, previous DENV unspecific antibodies may react with ZIKV antigen, but can only be detected in the lower dilutions (<1:1600). Later on, after 5 days post-symptoms, the titers of ZIKV antibodies increase during the secondary infection, but also do the DENV antibodies. This can be observed in Group IV. All samples were positive for DENV and ZIKV by RVNT_90_. Interestingly, the traditional ZIKV PRNT_90_ was not able to detect ZIKV antibodies in 88.2% of the samples, but using RVNT_90_ was possible to titer ZIKV antibodies in all of them. In addition, 23.5% (*n* = 4) of Group IV samples have ZIKV titer higher than DENV titer but it is not correlated to the time of disease (Table 1).

### 2.3. RVNT

**Figure 1:**
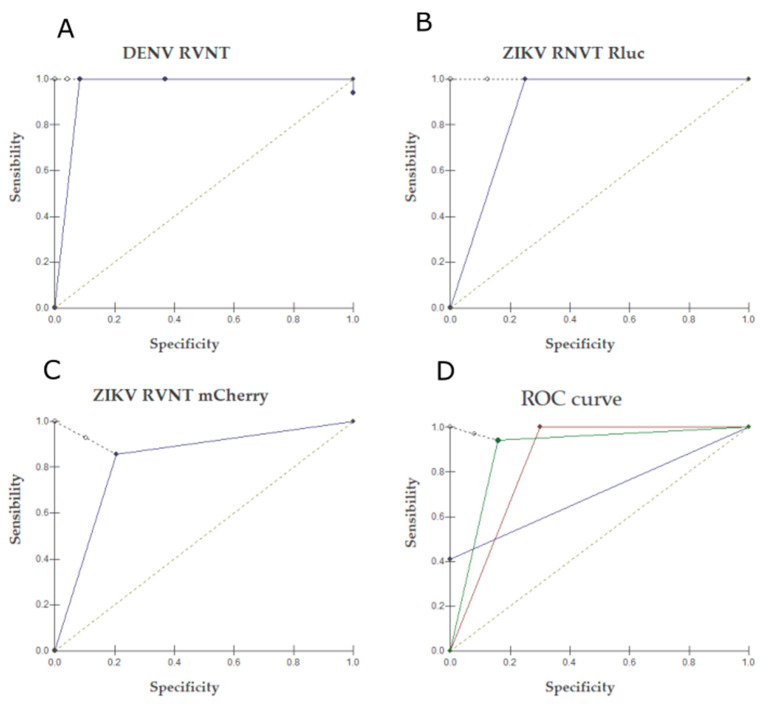
ROC curves comparing the performance of the DENV and ZIKV RVNT assays with the conventional PRNT. (**A**) DENV RVNT Rluc versus PRNT; (**B**) ZIKV RVNT Rluc versus PRNT; (**C**) ZIKV RVNT mCherry versus PRNT. (**D**) Comparison between ZIKV RVNT assays and the ZIKV PRNT using ZIKV diagnostic results (IgM-ELISA) as query.

For both DENV and ZIKV RVNT, (RLuc and mCherry), the test cut offs were kept at 90%, following PRNT criteria. However, the dilutions lower than 1:100 were defined as unspecific reactions since RVNT has been shown higher sensitivity compared to PRNT [23] due the high cross reaction observed among flavivirus. To calculate the RVNT_90_ assays’ sensitivity and specificity, all samples were also tested by traditional PRNT as the gold-standard test. For Dengue, DENV Rluc assay had a sensitivity and specificity equal to 100% and 78.3%, respectively, and showed 93.3% of accuracy. The ROC curve showed that DENV RVNT Rluc had a similar performance to PRNT (Figure 1A).

## 3. Discussion

The arbovirus circulation in Brazil has been constantly changing throughout the last decades, with successive epidemics, co-circulation of one or more serotypes of DENV [24,25], reemergence of (YFV) [26,27], and the introduction of ZIKV [24,28,29,30,31] and Chikungunya viruses [32,33]. Consequentially, an alarming increase has been noted in the number of people that have been affected. Because these viruses have similar symptoms, the laboratorial tools are essential to distinguish them. However, for flavivurus, the diagnosis is a huge challenge. A significant cross-reaction is observed using the traditional methods, such as Hemaglutition Inhibition (HI) and Mac-ELISA, leading to a high number of cases that remain without conclusion. For a long time, PRNT has been used for specific flavivirus diagnosis; however, the turnaround time to a final result can take a week. In this paper, it is shown that RVNT can efficiently replace the traditional PRNT in epidemic areas for flavivirus, reducing the result time to 3 days. 

**Figure 2:**
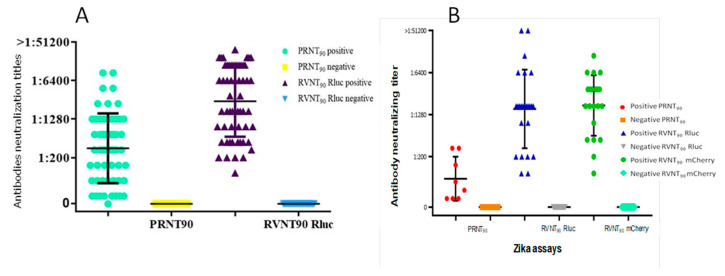
Neutralizing antibodies titers comparison. (**A**) DENV RVNT Rluc versus PRNT; (**B**) ZIKV RVNT Rluc and mCherry versus PRNT.

In samples from Groups I and II, the DENV RVNT was able to elucidate dengue diagnostics by detecting DENV specific neutralizing antibodies, with high sensitivity, specificity, and accuracy values, and similar performance as the PRNT (Figure 1A and Figure 2A). As for ZIKV differential diagnostics, there was low neutralization of ZIKV by unspecific antibodies, with more than 95% of samples with no cross-reaction detected between ZIKV and DENV (Figure 2B). The lack of ZIKV reactive antibodies produced in DENV infections has also been reported by Collins et al. 2017. These sera were harvested before the introduction of ZIKV in Brazil [24,34]; therefore, the lack of ZIKV exposition leads to the absence of ZIKV-specific antibodies. These data show that there is a reduced chance of ZIKV false positive results in samples from patients that have been exposed to other flavivirus but are naïve for ZIKV, suggesting that the ZIKV RVNT assay specificity is high even in regions that are highly endemic for other flavivirus such as DENV but with no ZIKV circulation (Figure 1B,C) [17,23].

However, one should be extra careful when analyzing the ZIKV RVNT results of samples from regions where there is actually co-circulation of ZIKV and other flavivirus such as DENV, especially in ZIKV secondary infections. We observed that in samples harvested during the ZIKV epidemics (Groups III and IV), there was high cross-reactivity between ZIKV and DENV. Interestingly, the majority of these samples (11/17) showed high total antibody titers for flavivirus in the IH assay (data not shown). Taking into consideration that IgM titers from flavivirus infections can persist for a long time, ranging from 90 days for DENV [35] to as long as one year for WNV [36], it is possible that high IgM antibodies titers from previous flavivirus infections may be contributing to this cross-reactivity. We should not rule out the possibility that this cross-reactivity could also be enhanced by the higher sensitivity of the RVNT when compared to the traditional PRNT.

The samples from Group III are ZIKV acute samples (less than 5 days of symptoms’ onset) diagnosed by ZIKV RT-qPCR; thus, no detectable ZIKV neutralizing antibodies were to be expected from this sample set. However, we observed that some samples (5/19) were positive for RVNT, whereas all samples were negative for the traditional PRNT (Table 1). Given the notoriously cross-reaction between flavivirus, these positive samples may be an unspecific reaction from cross-reactive DENV antibodies, as all these five samples also were positive for the DENV RVNT. Although unlikely, we should also not rule out the possibility that due to the ZIKV Rluc RVNT higher sensitivity as compared to the traditional PRNT (Figure 1D), the RVNT could be detecting ZIKV antibodies earlier in the infection when compared to PRNT. However, to evaluate this hypothesis, a different sample set of acute and convalescent samples with no history of previous flavivirus infection is needed.

If we now look specifically at the samples from Group IV, which contains only convalescent sera with more than five days of disease onset, it is extremely hard to identify ZIKV-specific results due to the mass cross-reaction with DENV antibodies that may be occurring given the results of the DENV RVNT. Even so, if we look at each sample individually, the ZIKV antibodies titers seem to be lower than the DENV antibodies titers, especially in the initial stages of disease. One possible solution for this problem is to request another sample collected later in the convalescent phase (more than 15 days) when the ZIKV antibodies titer might be higher than the previous DENV antibodies, as observed with sample 69 (Table 1), collected 50 days after disease onset. In fact, this increase of ZIKV antibodies titer collected later has been reported in a previous study using mother and neonate samples infected by ZIKV [35,37].

One of the PRNT’s disadvantages in flavivirus epidemic areas is the need of at least two samples to define a case and in most of the cases, the neutralizing antibodies can only be detected around 14 days after disease onset. In an experimental infection with non-human primates, using ZIKV RNVT90 mCherry to evaluate the efficacy of ZIKV vaccine candidate, Shan et al., 2017 [18] showed that this assay could detect ZIKV antibodies in the later viremic period. Here, we used samples with less than 5 days of the symptoms’ onset. This period is still early to define a conclusion for human samples showing secondary infections by flavivirus. Based on these results, we noticed that immune response profile of Brazilian population has been changed after ZIKV introduction.

Since the PRNT has been widely used to confirm MAC-ELISA results and exclude other flaviviruses, it would be invaluable to establish a RVNT panel, using flaviviruses of most medical importance, including ZIKV, DENV YFV, Saint Louis virus (SLV), West Nile virus (WNV), and Rocio virus (ROCV). In the case of DENV, where the co-circulation of viral serotypes is frequent, one could either choose the most predominant serotypes [17,38] or use DENV-2 as a standard, since it shows higher amino acidic relationship among all the other DENV serotypes, based on the E protein [39]. It would be interesting to investigate more components involved in the RVNT performance, the importance of both IgM and IgG in the cross-reactivity, and the evaluation of other arbovirus by RVNT in endemic areas.

## 4. Materials and Methods

### 4.1. Specimen Selection

This study was conducted by Instituto Evandro Chagas (IEC), Ananindeua, Para State, Brazil and University of Texas Medical Branch, Galveston, Texas, US (UTMB). All sera tested in this study were collected from patients who live in North or Northeast regions of Brazil, where co-circulation of ZIKV, DENV, yellow fever virus (YFV), and other flaviviruses has been detected. Furthermore, the secondary infection for all samples were confirmed by Hemagglutination Inhibition (HI) assay [40]. 

Seventy-five (75) serums were previously diagnosed to Dengue or ZIKV, based on authorized assays by Brazilian Minister of Health and PAHO (protocol number 3.601.702) and following the flowchart for arboviruses/ZIKV diagnosis in IEC (Figure S1). All of them were also tested by in-house MAC-ELISA IgM to other flaviviruses, such as YFV, Saint Louis Encephalitis virus (SLEV), West Nile virus (WNV), Ilheus virus (ILHV), and Rocio Virus (ROCV), showing negative results. Early acute patient sera, collected until 5 days after onset of disease symptoms, were previously tested by RT-qPCR [17], whereas later acute phase or convalescent patient samples were collected after 5 days of the onset of symptoms and tested by IgM-capture ELISA [41]. Samples were collected between 2000 and 2016, before and after ZIKV outbreak in Brazil (Table 1).

### 4.2. Cells and Viruses 

Vero E6 and BHK-21 cells were purchased from the American Type Culture Collection (ATCC, Bethesda, MD, USA), and maintained in a high-glucose Dulbecco′s modified Eagle′s medium (DMEM) supplemented with 10% fetal bovine serum (FBS; HyClone Laboratories, South Logan, UT) and 1% penicillin/streptomycin at 37 °C with 5% CO2. Infectious cDNA clone pFLZIKV were constructed previously and used to produce Renilla luciferase ZIKV, mCherry ZIKV (ZIKV Cambodian strain FSS13025) [20,42]. Renilla luciferase DENV-2 (strain NGC) was previously described by Zou et al., 2011. cDNA plasmids were used to in vitro transcribe genomic RNAs. Reporter ZIKV RNA transcripts were transfected into Vero cells, whereas reporter DENV-2 RNA were transfected in BHK-21 cells. To eliminate its interference with luciferase signal measurement, luciferase ZIKV and DENV-2 transfected cells were cultured in DMEM without phenol red. On day 10 (ZIKV) and 6 (DENV) post-transfection, culture fluids were collected and quantified for viral titers using an immuno-staining focus assay and plaque assay, respectively, as previously reported [42]. The three reporter genes were engineered at the beginning of the viral genome open-reading-frame, as detailed elsewhere [20,42,43]. For the standard PRNT assay, we used ZIKV Puerto Rico strain PRVABC59 and DENV-2 New Guinea (NGC) strain.

### 4.3. Plaque Reduction Neutralization Test 

Standard PRNT was performed according to Castanha et al., 2013 [44]. Virus dilutions of Puerto Vero cells and BHK cells concentration were 2 × 10^5^ cells/mL in a high-glucose Dulbecco′s modified Eagle′s medium (DMEM) supplemented with 10% fetal bovine serum (FBS; HyClone Laboratories, South Logan, UT, USA) and 1% penicillin/streptomycin at 37 °C with 5% CO_2_. In each 24-well plate, a single serum sample was tested in duplicate with DMEM 2% FBS and 1%P/S from 1:20 to 1:20480 dilution. After incubation, challenge, and infection, both for 1 h at 37 °C with 5% CO2, 500 µL of 0.8% methyl cellulose overlay was added. After 4 days the incubation, plates were fixed with 3.7% formaldehyde for 30 min and stained with 1% crystal violet for 5 min. The neutralization titers were determined by plaque quantity formed in controls wells (without antibody). The serum dilution that inhibited at least 90% of the tested virus inoculum (PRNT90) was considered the antibody titer [23,45].

### 4.4. Reporter Virus-Based Neutralization Assay 

Reporter ZIKV and DENV-2 containing a Renilla luciferase (Rluc) gene and reporter ZIKV containing mCherry (mCh) were used to measure the neutralization titers of patient sera against ZIKV or DENV-2, according to Shan et al., 2017 [21]. Briefly, Vero cells (1.5 × 10^4^ cells per well) were seeded into either 96-well white plate for Rluc or 96-well black plate for mCh (Corning Costar, St. Louis, MO, USA) one day prior to infection. Patient sera were initially diluted as 10-fold in a phenol red-free DMEM medium (ThermoFisher Scientific, Sugar Land, TX, USA) containing 2% FBS and 1% penicillin/streptomycin, followed by 2-fold serial dilution (1:100 to 1:51,200). Thirty microliters of each serum dilution were mixed thoroughly with 30 μL reporter ZIKV or DENV-2 and incubated at 37 °C for 1 h to form antibody-virus complexes. Afterwards, 50 μL serum-virus mixtures were inoculated onto the Vero cell monolayer (containing 50 μL phenol red-free DMEM medium with 2% FBS and 1% penicillin/streptomycin). The plate was incubated at 37 °C for 24 h (Rluc) or 48 h (mCh). The intracellular luciferase signals were measured using ViviRen substrates (Promega, Madison, WI, USA) and the mCherry fluorescence signals were measured directly on Cytation^5^ Cell Imaging Multi-Mode Reader (Biotek, Winooski, VT, USA) according to the manufacturer′s instructions. A medium containing the same amounts of reporter ZIKV or DENV-2 but without specimen serum was used as a non-treatment control and wells containing non-infected cells were used as non-signal controls for read background normalization for both luciferase and mCherry. Luciferase and mCherry signals from the non-treatment controls were set at 100%, whereas the signals from the non-signal controls were set at 0%. Luciferase and mCherry fluorescence signals from each diluted serum-treated samples were normalized to those from the non-treatment controls. A four-parameter sigmoidal (logistic) model in the software GraphPad Prism 7 was used to calculate the neutralization titers that suppressed 90% of the luciferase or mCherry signals of the non-treatment control RVNT90.

### 4.5. Statistical Analysis

The results were analyzed with the GraphPad Prism v7.02 software and Bioestat 5.3 [46]. Sensitivity, specificity, and accuracy for mCherry ZIKV, Rluc ZIKV, and Rluc DENV RVNT were calculated using the conventional PRNT90 assay as reference. In addition, sensitivity, specificity, and accuracy was calculated for mCherry ZIKV using Rluc ZIKV as reference [21]. Additionally, we calculated the ROC curve and McNemar test analysis was performed to compare neutralization techniques. All analyses used a *p*-value of <0.05 for statistical significance.

## 5. Conclusions

Finally, RVNT90 was not able to define a final diagnostic conclusion, but RVNT could be a useful tool to be included in the diagnostic algorithm of ZIKV and DENV alongside with clinical and epidemiologic data associated with MAC-ELISA results as indicative of recent infection. This could solve the cases of primary infection. As for the cases of secondary infection, a second sample collected later to evaluate seroconversion might be necessary especially in regions where the differential diagnosis represents a challenge. 

## Figures and Tables

**Table 1:** Samples per group with time of disease and the PRNT and RVNT antibodies titers.

	Samples				PRNT_90_		RVNT_90_		
Group	ID	#	Year	Time of Disease	ZIKV	DENV2	Rluc DENV2	Rluc ZIKV	mCherry ZIKV
	H726608	1	2007 ^a^	7 days	Neg	1:320	1:25,600	Neg	Neg
	H769882	2	2010 ^a^	4 days	Neg	1:20	1:400	Neg	Neg
	H769929	3	2010 ^a^	4 days	Neg	Neg	Neg	Neg	Neg
	H770060	4	2010 ^a^	2 days	Neg	Neg	1:200	Neg	Neg
	H770220	5	2010 ^a^	3 days	Neg	Neg	Neg	Neg	Neg
	H773035	6	2010 ^a^	3 days	Neg	1:80	1:1600	Neg	Neg
	H773052	7	2010 ^a^	4 days	Neg	1:20	1:12,800	Neg	Neg
	H773054	8	2010 ^a^	3 days	Neg	1:20	1:400	Neg	Neg
	H773564	9	2010 ^a^	4 days	Neg	Neg	Neg	Neg	Neg
I	H773565	10	2010 ^a^	4 days	Neg	1:320	1:12,800	Neg	Neg
	H773582	11	2010 ^a^	4 days	Neg	Neg	Neg	Neg	Neg
	H774749	12	2010 ^a^	5 days	Neg	1:160	1:3200	Neg	Neg
	H775262	13	2010 ^a^	<5 days	Neg	Neg	Neg	Neg	Neg
	H775844	14	2010 ^a^	<5 days	Neg	Neg	Neg	Neg	Neg
	H775845	15	2010 ^a^	<5 days	Neg	Neg	Neg	Neg	Neg
	H775848	16	2010 ^a^	<5 days	Neg	Neg	Neg	Neg	Neg
	H775852	17	2010 ^a^	<5 days	Neg	Neg	Neg	Neg	Neg
	H775853	18	2010 ^a^	<5 days	Neg	1:80	1:400	Neg	Neg
	H775854	19	2010 ^a^	<5 days	Neg	Neg	Neg	Neg	Neg
	H775862	20	2010 ^a^	<5 days	Neg	Neg	Neg	Neg	Neg
	H627940	21	2000 ^a^	>5 days	Neg	1:640	1:3200	Neg	Neg
	H632195	22	2000 ^a^	>5 days	Neg	1:320	1:3200	Neg	Neg
	H674584	23	2004 ^a^	>5 days	Neg	1:20	1:200	Neg	Neg
	H738095	24	2007 ^a^	>5 days	Neg	1:80	1:400	Neg	Neg
	H739187	25	2007 ^a^	6 days	Neg	1:320	1:12,800	Neg	Neg
	H739983	26	2008 ^a^	>5 days	Neg	1:640	1:6400	Neg	Neg
	H787665	27	2012 ^a^	14 days	Neg	1:10,240	1:25,600	Neg	Neg
	H788930	28	2012 ^a^	>5 days	Neg	1:640	1:6400	Neg	Neg
	H789010	29	2012 ^a^	>5 days	Neg	1:5120	1:25,600	Neg	Neg
II	H789197	30	2012 ^a^	>5 days	Neg	1:10,240	>1:51,200	1:200	1:200
	H789912	31	2012 ^a^	>5 days	Neg	1:160	1:800	Neg	Neg
	H789990	32	2012 ^a^	>5 days	Neg	1:640	1:12,800	Neg	Neg
	H789997	33	2012 ^a^	>5 days	Neg	1:2560	1:25,600	1:100	Neg
	H790260	34	2012 ^a^	>5 days	Neg	1:1280	1:6400	Neg	Neg
	H815485	35	2015	>5 days	Neg	1:20	Neg	Neg	Neg
	H817979	36	2015	>5 days	Neg	1:20	1:200	Neg	Neg
	H817981	37	2016	>5 days	Neg	1:1280	1:12,800	Neg	Neg
	H819129	38	2015	>5 days	Neg	1:160	1:1600	Neg	Neg
	H819519	39	2015	>5 days	Neg	1:320	1:1600	Neg	Neg
	H817986	40	2015	<5 days	Neg	Neg	Neg	Neg	Neg
	H819966	41	2015	3 days	Neg	Neg	Neg	Neg	Neg
	H820771	42	2015	1 day	Neg	1:320	1:1600	1:800	1:400
	H821519	43	2015	<5 days	Neg	1:160	1:1600	Neg	Neg
	H821557	44	2015	2 days	Neg	1:80	1:800	1:200	1:100
	H821585	45	2015	3 days	Neg	1:20	1:200	1:100	Neg
	H821735	46	2015	<5 days	Neg	Neg	1:400	Neg	Neg
	H821956	47	2015	2 days	Neg	Neg	Neg	Neg	Neg
III	H822137	48	2015	4 days	Neg	Neg	1:100	Neg	Neg
	H822215	49	2015	<5 days	Neg	1:80	1:400	Neg	Neg
	H822217	50	2015	<5 days	Neg	1:80	1:800	Neg	Neg
	H822226	51	2015	3 days	Neg	Neg	Neg	Neg	Neg
	H822604	52	2015	2 days	Neg	Neg	Neg	Neg	Neg
	H823608	53	2015	4 days	Neg	Neg	Neg	Neg	Neg
	H823390	54	2015	3 days	Neg	1:80	1:800	1:200	Neg
	H823594	55	2015	1 day	Neg	Neg	1:400	Neg	Neg
	H824562	56	2015	3 days	Neg	1:320	1:800	Neg	Neg
	H824564	57	2015	1 day	Neg	Neg	Neg	Neg	Neg
	H824708	58	2015 ^b^	4 days	Neg	Neg	1:1600	1:6400	1:6400
	H816971	59	2015	12 days	1:20	1:1280	1:12,800	1:1600	1:1600
	H819282	60	2015	>5 days	1:40	1:5120	1:25,600	1:6400	1:3200
	H819284	61	2015 ^b^	>5 days	1:80	1:1280	1:6400	>1:51,200	1:3200
	H819360	62	2015	8 days	Neg	1:640	1:12,800	1:1600	1:400
	H820967	63	2015	7 days	Neg	1:1280	1:12,800	1:1600	1:800
	H821489	64	2016	>5 days	1:320	1:2560	1:12,800	1:12,800	1:1600
	H821491	65	2015 ^b^	>5 days	Neg	1:160	1:800	1:1600	1:3200
	H822200	66	2015	>5 days	Neg	1:160	1:1600	1:1600	1:1600
	H822413	67	2015	>5 days	Neg	1:1280	1:6400	1:1600	1:6400
VI	H822540	68	2015	>5 days	1:20	1:640	1:6400	1:1600	Neg
	H823176	69	2015 ^b^	50 days	1:160	1:640	1:6400	>1:51,200	1:12,800
	H824696	70	2015	7 days	Neg	1:320	1:1600	1:200	1:400
	H825051	71	2015	11 days	Neg	1:640	1:12,800	1:1600	1:3200
	H826145	72	2016 ^b^	>5 days	Neg	1:20	1:400	1:1600	1:1600
	H828107	73	2016	>5 days	Neg	1:1280	1:25,600	1:1600	1:3200
	H828108	74	2016	>5 days	Neg	1:1280	1:6400	1:6400	1:6400
	H829179	75	2016	19 days	1:20	1:1280	1:6400	1:1600	1:3200

## Supplementary Materials

The following are available online at https://www.mdpi.com/article/10.3390/pathogens10070840/s1, Figure S1: Arbovirus diagnosis algorithm. Table S1. Contingency table with the RVNT assays diagnostics predictive values. All RVNT assays were evaluated in comparison to the reference assay PRNT.
