# Supplementary material for: Reporter Virus Neutralization Test Evaluation for Dengue and Zika Virus Diagnosis in Flavivirus Endemic Area
Source: Pathogens. 2021 Jul 3;10(7):840. doi: 10.3390/pathogens10070840 (PMC8308650; doi:10.3390/pathogens10070840)
Supplement: Supplementary file 1 [file pathogens-10-00840-s001.zip › pathogens-1219320-supplementary.pdf]

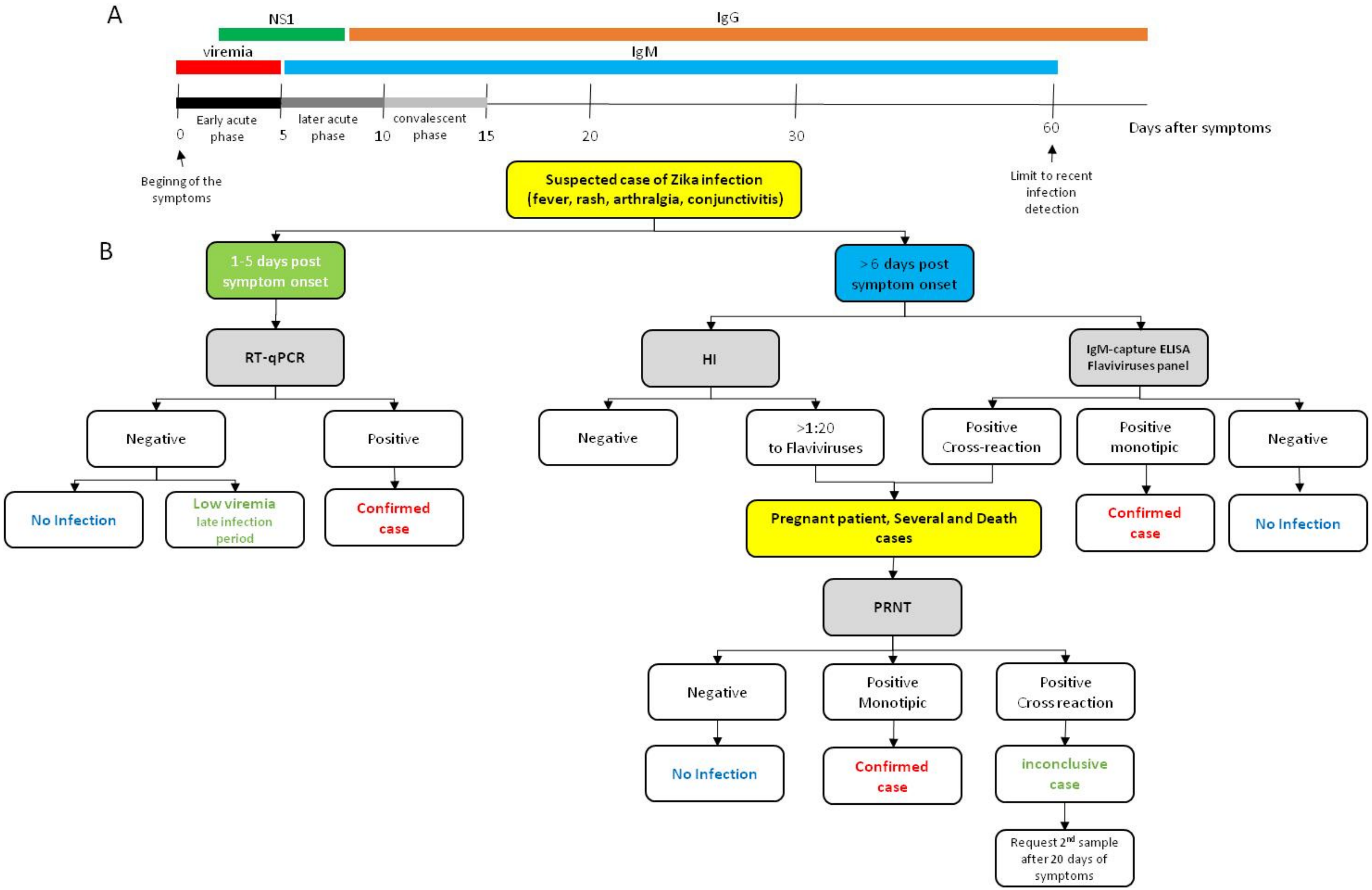

Figure S1. Arbovirus diagnostic algorithm. Diagnostic algorithm followed by the Department of Arbovirology and Hemorragic Fevers of Instituto Evandro Chagas, currently the national reference for arbovirus diagnostics in Brazil.

Table S1. Contingency table with the RVNT assays diagnostics predictive values. All RVNT assays were evaluated in comparison to the reference assay PRNT.

Query	**Tests**	PPV	NPV	**Sensitivity (%)**	**Specificity (%)**	**Accuracy (%)**	McNemar value
PRNT90	DENV RVNT$_{90}$ Rluc	91.2	100	100	78.3	93.3	0.218 ($p>0,05$)
	RVNT$_{90}$ Rluc	32.0	100.0	100.0	74.6	77.3	0.000 ($p<0,05$)
	RVNT$_{90}$ mCherry	33.3	98.1	88,00	79.10	80.0	0.001 ($p<0,05$)
ZIKV Rluc	RVNT$_{90}$ mCherry	100.00	92.59	84.0	100.0	94.7	0.125 ($p>0,05$)
